# Emerging machine learning approaches to phenotyping cellular motility and morphodynamics

**DOI:** 10.1088/1478-3975/abffbe

**Published:** 2021-06-17

**Authors:** Hee June Choi, Chuangqi Wang, Xiang Pan, Junbong Jang, Mengzhi Cao, Joseph A Brazzo, Yongho Bae, Kwonmoo Lee

**Affiliations:** 1 Department of Biomedical Engineering, Worcester Polytechnic Institute, Worcester, MA 01609, United States of America; 2 Vascular Biology Program and Department of Surgery, Boston Children’s Hospital, Harvard Medical School, Boston, MA 02115, United States of America; 3 Data Science Program, Worcester Polytechnic Institute, Worcester, MA 01609, United States of America; 4 Department of Pathology and Anatomical Sciences, Jacobs School of Medicine and Biomedical Sciences, University at Buffalo, State University of New York, Buffalo, NY 14203, United States of America; 5 Present address. Department of Biological Engineering, Massachusetts Institute of Technology, Cambridge, MA 02139, USA.

**Keywords:** machine learning, live cell imaging, deep learning, phenotyping, cell motility, cell morphodynamics

## Abstract

Cells respond heterogeneously to molecular and environmental perturbations. Phenotypic heterogeneity, wherein multiple phenotypes coexist in the same conditions, presents challenges when interpreting the observed heterogeneity. Advances in live cell microscopy allow researchers to acquire an unprecedented amount of live cell image data at high spatiotemporal resolutions. Phenotyping cellular dynamics, however, is a nontrivial task and requires machine learning (ML) approaches to discern phenotypic heterogeneity from live cell images. In recent years, ML has proven instrumental in biomedical research, allowing scientists to implement sophisticated computation in which computers learn and effectively perform specific analyses with minimal human instruction or intervention. In this review, we discuss how ML has been recently employed in the study of cell motility and morphodynamics to identify phenotypes from computer vision analysis. We focus on new approaches to extract and learn meaningful spatiotemporal features from complex live cell images for cellular and subcellular phenotyping.

## 1. Introduction

A primary goal of biology is to understand the phenotypic characteristics of organisms and their underlying mechanisms. Indeed, biology is a branch of natural science that has historically focused on the study of differences in organismal traits or phenotypes (appendix A). The careful observation of phenotypes has traditionally been important in both medicine and agriculture. Prior to the advent of modern medicine, medical diagnosis focused on carefully detecting abnormal phenotypes and treating the associated diseases. The recent advancement of high-throughput tools has accelerated phenotyping at the molecular, cellular, and tissue levels [1]. Advances have been made in many areas by employing state-of-the-art genomic and imaging technologies, including medical diagnosis [2–5], drug discovery [6–13], agriculture [14–16], and bioproduction [17, 18].

Conventionally, phenotypes are characterized by static information such as cell morphology, protein abundance, and localization. Living organisms, however, dynamically react to wide ranges of everchanging environments by adapting their biochemical, physical, and morphological characteristics to new environments. Cellular dynamic responses occur on various timescales: biochemical signaling within seconds, transcriptional changes from minutes to hours, and differentiation and division from hours and days [19]. For example, the protein level of p53, a tumor suppressing transcription factor that controls cell division and cell death, displays dynamic oscillations in response to DNA damage to mitigate the irreversible effects of perpetual activation of p53 target genes [20, 21]. Cells also dynamically change their morphology within seconds to hours in response to environmental cues [22]. It is increasingly clear that static phenotypes are limited when investigating such dynamic processes and should be complemented by temporal organismic behaviors called ‘dynamic phenotypes’ [23].

Since cell morphology and motility reflect the physiological and signaling states of a cell [24–26], efforts have long been underway to analyze the dynamics of cell morphology (morphodynamics) as well as motility and locomotion in a quantitative manner using live cell imaging [27–29]. However, phenotypic heterogeneity, where multiple phenotypes coexist at subcellular [30–32], cellular [33–35], and multicellular [36, 37] levels, hinders the task of phenotyping from a large and high-dimensional live cell dataset. Moreover, highly dynamic new phenotypes can emerge depending on environmental conditions and developmental age [38]. Therefore, the heterogeneity of cellular and subcellular dynamics has been a significant challenge for the quantitative identification of dynamic phenotypes.

Computer vision and machine learning (ML) have been employed to extract quantitative information from cell images and have become key tools for identifying cellular phenotypes [39]. There are existing tools that aid computational image analysis, including morphological profiling (CellProfiler [40] and PhenoRipper [41]), the supervised learning of cellular phenotypes (CellClassifier [42]), and the discovery of phenotypes in high-content imaging data (advanced cell classifier [43]). While these methods have been used to distinguish between normal and cancer cells [44, 45], these tools have been limited to static cell image datasets. Computer vision and ML enable us to identify previously unknown dynamic phenotypes that cannot be detected by the human eye. These new technologies are capable of unraveling phenotypic heterogeneity and opening a new avenue for defining phenotypes at unprecedented spatial and temporal resolutions. In this review, we focus on how ML can address the issues of phenotypic heterogeneity within live cell image datasets and identify spatiotemporal phenotypes of cell motility and morphodynamics.

## 2. Feature extraction in machine learning to recognize phenotypes

In recent years, ML has proven to be instrumental in biomedical research, allowing scientists and clinicians alike to implement sophisticated computation in which computers learn and effectively perform specific tasks from biologically relevant datasets with minimal human instruction or intervention. ML can be mainly categorized into supervised and unsupervised learning. Supervised learning requires labeled datasets and discovers the relationship between inputs and outputs of ML systems. Unsupervised learning utilizes data representations as input and discovers the internal structures of data. In terms of phenotyping, supervised learning can be used to classify data into known phenotypes, and unsupervised learning can be used to discover previously unknown phenotypes. Conceptually, both supervised and unsupervised learning consist of feature extraction and optimization, which can include classification, regression, or clustering. After features are extracted from the data, supervised learning can be performed for classification or regression, or unsupervised learning can be performed for data clustering. The goal of these procedures is to optimize the objective functions related to the criteria for given ML tasks.

Raw datasets from live cell imaging inherently have high dimensionality (a time-lapse image set with 100 × 100 pixels and 10 timeframes has 10^5^ dimensions). This leads to the ‘curse of dimensionality’ [46, 47], wherein the volume of data space becomes exponentially large as the dimensionality increases. This makes the data distribution too sparse, ultimately hindering computational algorithms from reaching statistically significant results. Therefore, for the effective phenotyping of high-dimensional datasets, it is necessary to project raw data onto low-dimensional space while retaining intrinsic dimensions. The goal of feature extraction is to represent raw data effectively using relevant features with lower dimensions. The new representations derived from raw datasets have less noise, redundancy, and dimensionality than the original dataset, making them more informative and beneficial to subsequent computational processes. Therefore, one of the most important steps to determine the success of ML applications is feature extraction.

**Figure 1.**
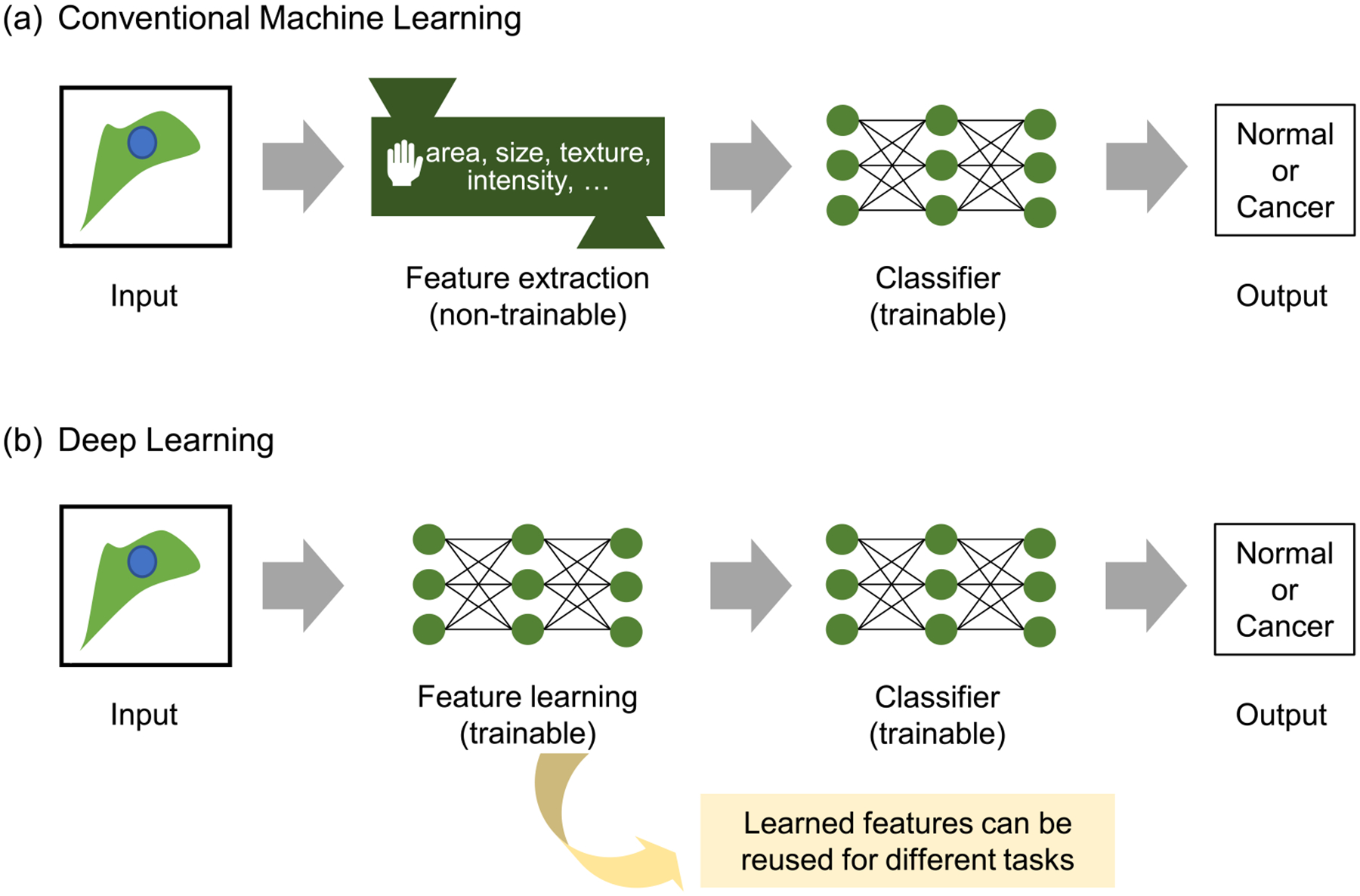
Comparison between conventional machine learning and deep learning. (a) In conventional machine learning, we need to extract handcrafted features from raw data. These features are used to train the classifier. (b) In deep learning, feature learning and classifier training are performed end-to-end. After the training, the trained feature extractor can produce meaningful features, which can be reused for different tasks, including unsupervised phenotyping.

In traditional ML contexts (figure 1(a)), manually selected features are used for dimensionality reduction. This process, however, is very time consuming and requires considerable human effort. An alternative method for feature extraction is feature learning, in which computers learn features directly from the data with less human instruction (figure 1(b)). Deep learning (appendix B) offered a breakthrough in feature learning, wherein deep neural networks (DNNs) can learn the relevant features automatically and directly from raw data through multiple hidden layers [48]. Since the deep learning approach utilizes all the information from raw data, it provides more comprehensive features that human intuition cannot offer, while the manually selected features used in traditional ML are generally more interpretable and related to domain knowledge. Autoencoders (AEs), based on deep learning, have been widely used for feature learning [49] because they reproduce the input of the AE while limiting the number of hidden units. The values from these hidden units can serve as the learned features from the data. The learned features from one domain can be used in another domain by transfer learning, which extracts feature information from input data using networks pretrained on different but related domains [50, 51]. Convolutional neural network (CNN) models pretrained with numerous ordinary images available on ImageNet [52] have been shown to generate highly effective features in many image-related ML tasks [53–56].

## 3. Phenotypes of cellular motility and morphodynamics at various spatiotemporal scales

Cells and subcellular structures constantly undergo heterogeneous morphological changes over various spatiotemporal scales. Advances in fluorescence microscopy have allowed researchers to acquire an unprecedented amount of live cell image data at high spatiotemporal resolutions, which has revealed a massive amount of heterogeneity. Current image analysis tools, however, usually have limited capacity for phenotyping cellular and subcellular behaviors from heterogeneous live cell image datasets. ML and deep learning are being increasingly employed to extract spatiotemporal features from live-cell imaging data and identify their phenotypes to better understand the underlying biological mechanisms. In this section, we briefly review recent efforts to understand the diverse dynamic phenotypes of cell motility and cellular morphodynamics on various length and time scales.

### Cell motility.

Cell motility is an essential process for various physiological and pathophysiological processes such as development, immune responses, wound healing, angiogenesis, and cancer metastasis. Cell motility occurs on time scales from hours to days and has long been a subject of study in the field of quantitative cell biology. Previously, cell migration trajectories were studied using simple random walk models [28, 29]. However, it is increasingly recognized that there exists significant cell-to-cell variability in motility speed [57], and cell motility has multiple phenotypes representing unique cellular states. Several ML frameworks for time-lapse live cell images have been developed to characterize numerous motility phenotypes using the unsupervised learning of single-cell motility [58–60] and collective cell migration [36, 61]. A recent study suggested that motility phenotypes in muscle stem cells (MuSCs) in mice represented the intermediate steps of MuSC differentiation [59]. The motility phenotyping of retinal progenitor cells (RPCs) before mitosis allowed for the prediction of the fate of RPCs (self-renewing vs terminal division and photoreceptor vs nonphotoreceptor progeny) [62]. The distance traveled by bone marrow mesenchymal stem cells was correlated with their adipogenic, chondrogenic, and osteogenic differentiation potentials [63]. The motility speed of human osteosarcoma cells can distinguish between a dormant nonangiogenic phenotype and an active angiogenic phenotype [64]. Knockout of a breast cancer oncogene, lipocalin 2, in human triple-negative breast cancer cells significantly reduced the motility speed and the migration distance [65].

### Cellular morphodynamics.

Leading edges (protrusive plasma membranes at the front of the cell) of migrating cells display significant spatiotemporal heterogeneity [27, 30]. Although these morphodynamic events occur on time scales from minutes to hours, which is much faster than cell motility, the morphology of motile cells by itself tells us about the biochemistry and mechanics underlying cell motility [66]. Recent ML analyses of live cell images revealed the relationships between cell morphodynamics and motility as follows. The morphological changes over second timescales enabled the prediction of migratory behaviors over minute timescales in *Dictyostelium* [67]. The morphological coordination between protrusion and retraction determines metastatic potency [68] and governs switching between ‘continuous’ and ‘discontinuous’ mesenchymal migratory phenotypes [69]. Morphodynamic phenotypic biomarkers together with migratory information can be used to evaluate the metastatic potential of breast and prostate cancer cells [70]. The neuronal growth cone is an essential structure for guiding axons to their targets during neural development. It exhibits complex and rapidly changing morphology, and the morphology of neuronal growth cones is highly correlated with neurite outgrowth [71]. The significance of morphodynamic phenotypes is not limited to cell motility. For example, the time-series modeling of live cell shape dynamics can reveal differential drug responses in breast cancer cells [72]. The local protrusion patterns of leukocytes can inform immune responses [73]. Finally, the morphodynamics of hematopoietic stem and progenitor cells (HSPCs) can predict the lineage before three generations [74].

### Cell motility and morphodynamics in 3D environments.

A more recent study emphasized the importance of understanding cell motility in 3D cultures due to its physiological relevance and the advent of 3D cultures [75] and light sheet microscopy [76]. The persistent random walk model used for 2D motility is not suitable for modeling 3D motility since 3D motility is temporally coupled and anisotropic [77]. There are also significant technical challenges in assessing 3D cell morphology in a quantitative manner compared to 2D morphology [78]. The 3D morphological features of cancer cells derived from quantitative phase contrast microscopy can classify healthy, cancer, and metastatic cells [79]. A 3D image analysis of endothelial cell branching in 3D collagen gels revealed the role of myosin II in shape control [80]. Recently, an ML-based 3D morphological motif detector, u-shape 3D, was developed to identify lamellipodia, filopodia, pseudopodia and blebs in 3D live cell images, clearing a path for 3D subcellular morphodynamic phenotyping [81].

### Subcellular cytoskeleton dynamics.

The cytoskeleton orchestrates cellular morphodynamics and motility. Therefore, characterizing the heterogeneity of cytoskeletal dynamics at subcellular levels can reveal the underlying mechanism for cell motility and morphodynamics. The actin cytoskeleton directly affects cellular morphology via actin remodeling. Lamellipodia are composed of actin network structures at the leading edge of a motile cell, which provide strong force generation for cell shape changes and motility. Quantitative fluorescence speckle microscopy [82] and local sampling strategies [83, 84] have been extensively used to probe lamellipodial dynamics in cell protrusions. Furthermore, the leading edge dynamics of lamellipodia were shown to have distinct subcellular protrusion phenotypes with differential recruitment of VASP and Arp2/3 [30]. Extended lamellipodia promoted by hyperactivation of Rac1 via the P29S mutation promotes the proliferation of melanoma cells [85]. Filopodia are finger-like protrusive structures containing thin actin bundles that play a role as sensory organelles for cell migration. Several computational platforms for the analysis of filopodial dynamics have been developed, including cellGeo [86], filopodyan [87], filoQuant [88], and GCA [71]. In neuronal growth cones, there are multiple filopodial phenotypes whereby Ena and VASP play differential roles in associated filopodial dynamics [87]. Additionally, the increased density of filopodia in breast cancer cells promotes higher invasiveness [88].

## 4. Strategies for spatiotemporal feature extraction

With the advancement of live cell imaging techniques, various computational strategies have been employed to extract both spatial and temporal features to characterize new phenotypes. As discussed before, feature extraction is a critical step for successful ML-based phenotyping because the nature of the extracted features from raw data determines the phenotyping. In this section, we discuss analytical strategies for feature extraction to identify dynamic cellular phenotypes in detail. We first focus on handcrafted feature extraction. Then, we discuss the application of emerging feature learning-based methods using deep learning.

### 4.1. Handcrafted feature extraction

**Figure 2.**
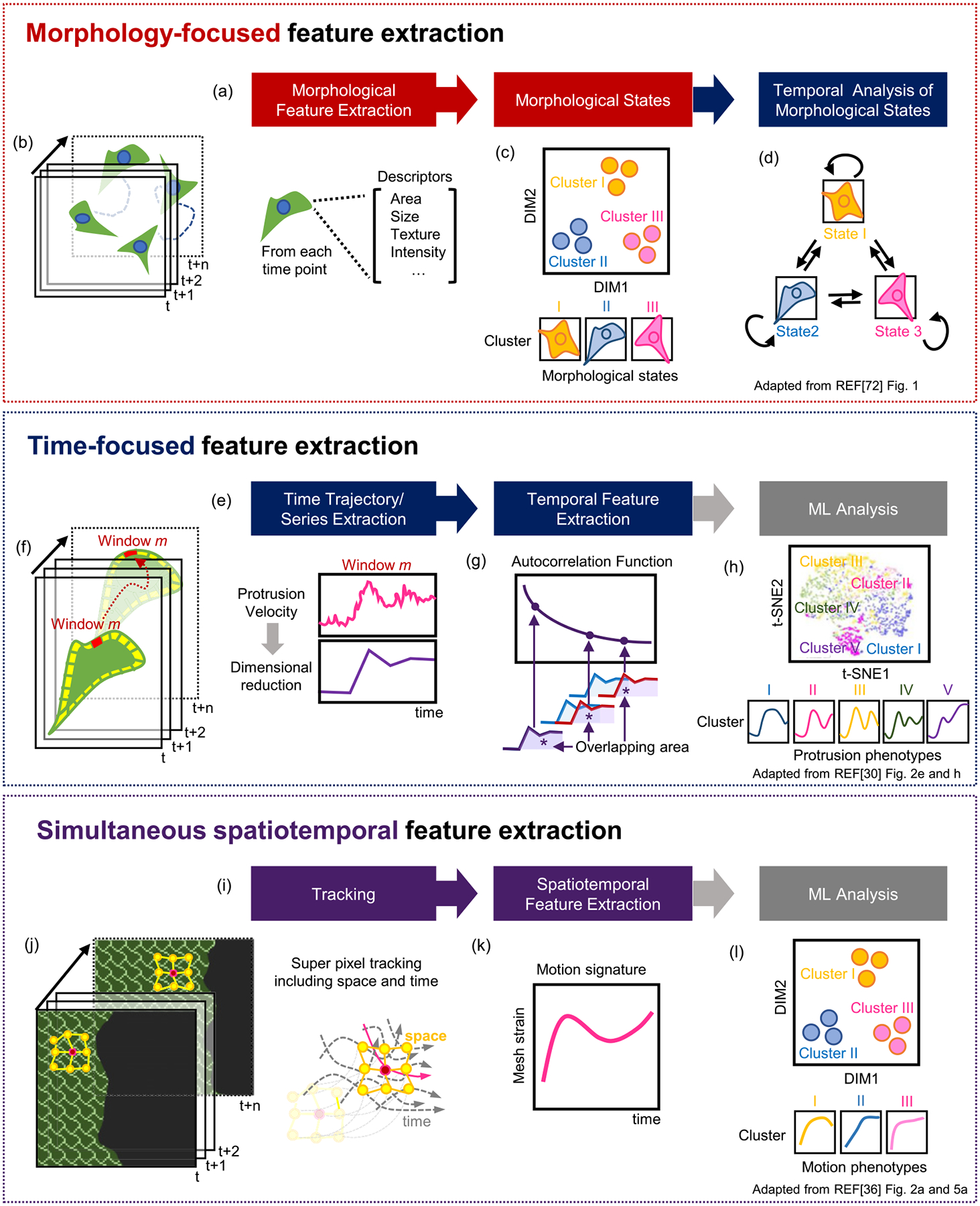
Feature extraction-based phenotyping of cell motility and morphodynamics. (a) Phenotyping based on morphology-focused feature extraction. (b) Cellular morphology at each timepoint. (c) Identification of morphological states by dimensional reduction of morphological features. (d) Temporal transition of morphological states. (e) Phenotyping based on time-focused feature extraction. (f) Examples of subcellular protrusion time series. (g) Extraction of autocorrelation function (ACF) temporal features. (h) Subcellular protrusion velocity phenotypes. (i) Phenotyping based on simultaneous spatiotemporal feature extraction. (j) An example of tracking cells in collective migration. (k) Methods for spatiotemporal feature extraction. (l) Phenotypes of the strain curves from collective cell migration. Panels (c) and (d) are adapted with permission from figure 1 in reference [72], Oxford University Press. Panel (h) is adapted from figures 2(e) and (h) in reference [30]. Panels (j)–(l) are adapted from figures 2(a) and 5(a) in reference [36]. Panels (h) and (j)–(l) are licensed under (CC-BY-4.0).

The overall strategy defined here can be largely grouped by which type of feature is the focus for identifying phenotypes as follows (figure 2): (i) morphology-focused extraction, followed by temporal analysis, (ii) time-focused feature extraction, and (iii) simultaneous spatiotemporal feature extraction. Below is a more detailed description of representative analytical processes in defining cellular phenotypes in each category.

#### Morphology-focused feature extraction.

In this category (figure 2(a)), rich morphological features are first extracted at each time point (figure 2(b)); then, the feature dimensions are often reduced by principal component analysis [89] (PCA, reducing the dimensionality of data while preserving the information of data as much as possible), and morphological states are identified (figure 2(c)). The subsequent temporal analyses are performed by ML, such as time-series modeling, clustering, and classification. These types of analyses usually assume that cell morphology dictates distinct cellular states and then investigate how the morphological states evolve over time (figure 2(d)).

Gerlich’s group pioneered the development of ML frameworks for cellular morphodynamics in mitosis. Their overall approach focused on accurate morphological phenotyping by taking advantage of temporal information. Held *et al* developed a supervised ML framework, termed CellCognition [90], to classify complex cellular dynamics through morphologically distinct cell states (interphase, six different mitotic stages, and apoptosis) combining support vector machine [91] (SVM, see appendix C) classification with a hidden Markov model [92] (HMM, see appendix C). They extracted 186 quantitative features of texture and shape from the confocal images of live HeLa cells stably expressing the chromatin marker H2B-mCherry, followed by SVM training for the classification of the cell states. Thereafter, they trained an HMM to take advantage of the temporal context of the cellular states, which corrected the misclassification occurring during the state transition. Zhong *et al*, in the same group, developed an unsupervised ML approach to identify the different cellular stages in mitosis without user annotation [93]. The same morphological features were reduced by PCA, and then temporally constrained combinatorial clustering was applied to make temporally linked objects cluster together, producing results consistent with the user annotation. This avoids expensive user annotation and facilitates high-throughput image-based screening.

To analyze morphodynamics in cell motility, Godonov *et al* developed an unsupervised ML method, the SAPHIRE (stochastic annotation of phenotypic individual-cell responses) framework [72], wherein 18 morphological features, including area, perimeter, equivalent diameter, major/minor axis length, eccentricity, solidity, extent, convex area, axis ratio, circularity, waviness, geodesic diameter, and convex diameter, were extracted for each cell object at a specific temporal point. The high dimensionality of the extracted features is then reduced to low-dimensional space by PCA, and distinct shape states are determined by clustering. Thereafter, they applied an HMM to the time trajectories of these PCA-reduced features. Similarly, with the previously mentioned works, they also considered that cell morphology represents cellular states, and the HMM was applied to study the dynamics of the transition between morphological states. The temporal features extracted from the HMM can reveal more refined drug effects than the morphological features alone, which can be used to dissect the heterogeneity in cellular drug responses.

Morphodynamic phenotypes were also studied in the context of epithelial-to-mesenchymal transition (EMT) by Wang *et al* [94]. Using 150 points on cell outlines as cellular morphological features, they extracted Haralick features [95] (quantifying texture information from images) from fluorescently tagged endogenous vimentin, which is an EMT marker. After tracking these features over time during EMT, the acquired time series were projected onto a 2D space using a nonlinear dimensionality reduction technique, t-SNE [96] (t-distributed stochastic neighbor embedding, assigning pairs of similar data with high probabilities of being neighbors in low dimensional space), and they found two clusters using *k*-means clustering [97] (grouping unlabeled data into *k* clusters by assigning them to nearest cluster means). In one cluster, the changes in vimentin Haralick features preceded those of cell morphology. In the second cluster, the vimentin Haralick and morphological features changed concertedly. Since they could not find these results using pseudo-time analysis from the snapshot data, their live cell image analysis revealed the heterogeneity of EMT trajectories, which could not be obtained from static datasets.

The morphodynamic phenotyping of neuronal growth cones was studied using PCA in shape space, which revealed five to six basic shape modes of neuronal growth cone morphology [98]. For each growth cone mode, the autocorrelation function [99] (ACF, quantifying the similarity between a time series and its lagged one) and Fourier power spectrum were quantified to identify oscillatory phenotypes. It was found that the average growth rate was significantly correlated with the oscillation strength of specific growth cone modes.

#### Time-focused feature extraction.

Previous morphology-focused approaches have treated cell morphology as a readout of cell states. While this can be a faithful approximation, particularly in mitosis, it is also possible that temporal dynamics in cellular or subcellular processes can reflect their unique properties. Therefore, temporal phenotyping based on time-focused feature extraction could reveal new findings. To identify temporal phenotypes (figure 2(e)), we must extract time-series or trajectory data directly from live-cell time-lapse videos using image analysis (figure 2(f)), and then quantify specific temporal features from the time-series (figure 2(g)). If necessary, the dimensions of temporal features can be reduced by PCA. Thereafter, standard ML methods such as clustering or classification can be applied to characterize the temporal phenotypes (figure 2(h)). The identified temporal phenotypes can then be further studied in a spatial context. This approach has been applied in cell motility, protrusion, and endocytosis, as discussed below.

The initial efforts in this approach were focused on phenotyping cell motility based on cellular trajectories. Sebag *et al* [58] developed a generic unsupervised ML method termed MotIW (motility study integrated workflow) from high-throughput time-lapse image data. First, fifteen features were extracted from each cellular trajectory, including the diffusion coefficient and track entropy, other global features (such as convex hull area and effective path length), and average local features (including mean square displacement and mean signed turning angle). PCA was used for dimensionality reduction, and *k*-means clustering was applied. Using the Mitocheck dataset, they discovered eight motility phenotypes, but the biological meaning of each phenotype remains to be discerned. Kimmel *et al* [59] took a similar approach, termed heteromotility, that extracts motility features from time-lapse cell images, including distance traveled, turning, and speed metrics. Using heteromotility, they identified more detailed phenotypes because the approach included features with more complex motions, such as Levy flight-like motion features, fractal Brownian motion features, and autocorrelation functions for displacements. Subsequently, hierarchical clustering was performed with Ward’s linkage, which identified multiple motility phenotypes within the cell population. The application of heteromotility analysis to the MuSC system during activation revealed three distinct MuSC phenotypes, and the motility phenotypes of activated MuSCs sequentially led to the motility phenotypes of muscle progenitor myoblasts, suggesting that dynamic phenotypes of cell motility can represent the intermediate steps of MuSC differentiation.

Multiple phenotypes exist not only at the single-cell level but also at the subcellular level. Therefore, dynamic phenotyping has been applied to subcellular leading-edge dynamics. Wang *et al* [30] developed an unsupervised ML framework, termed HACKS (deconvolution of heterogeneous activity in the coordination of cytoskeleton at the subcellular level) that deconvolves the heterogeneity of the subcellular protrusion at micron and minute scales. The images of the leading edge of PtK1 epithelial cells were segmented by multiple probing windows, and then the time series of protrusion velocities in each probing window were quantified (figure 2(f)). The ACF was calculated as a temporal feature of the protrusion velocity time series (figure 2(g)). Then, density peak clustering [100] (identifying cluster centers that are at local density maxima and away from other high-density regions) was applied to identify distinct subcellular protrusion phenotypes (figure 2(h)). HACKS identified distinct subcellular protrusion phenotypes hidden in highly heterogeneous protrusion activities, revealing the temporal coordination of Arp2/3 and VASP in accelerating protrusion phenotypes. This analysis also suggests that the unsupervised ML of cellular dynamics could dissect the underlying molecular mechanisms and drug responses obscured by heterogeneity. Li *et al* also analyzed the subcellular dynamics of leading-edge displacement using unsupervised ML and demonstrated that the subcellular phenotypes could be utilized for single-cell phenotyping [101]. They tracked and segmented target cells from the phase contrast microscopy images of lymphocytes. The local time series of edge displacement was extracted, and six-dimensional temporal features, such as temporal regularity, were quantified. Based on the local temporal features, the researchers further reduced the dimension of the vector to 2 with PCA, followed by applying *k*-means clustering. Three clusters of subcellular deformation patterns were identified, and the frequency of each cluster within the same cells was used for the feature of dynamic cellular morphology to perform supervised ML to classify ‘normal’ and ‘drastic’ cellular phenotypes.

Membrane trafficking, including endocytosis and exocytosis, is also a promising field where this ML application can make a significant contribution due to the multiple modes of endocytic and exocytic events [102, 103]. Wang *et al* [102] developed a ML method termed DASC (disassembly asymmetry score classification) applied to clathrin-mediated endocytosis (CME), that resolves aborted coats (ACs) from bona fide clathrin-coated pits (CCPs) based on single-channel fluorescent movies. They defined a clathrin disassembly risk function, which indicates the net risk for disassembly at every intensity-time state from the fluorescence time series. From this risk function, they quantified the features, including time-average, lifetime-normalized difference between the maximum and minimum value, and a modified skewness of the disassembly risk, which allowed accurate classification between ACs and CCPs. DASC can provide more accurate pictures of the progression of CME by deconvolving previously unresolvable ACs and CCPs.

#### Simultaneous spatiotemporal feature extraction.

In the previous ML approaches, spatial and temporal analyses were performed sequentially. However, when spatial and temporal processes are tightly interconnected with each other, it is desirable to consider spatiotemporal features simultaneously (figure 2(i)). This approach may preserve more information about spatiotemporal coordination than sequential procedures.

Spatiotemporal feature extraction is particularly relevant in collective cell migration, where neighboring cells constantly interact to migrate together. To study the propagation of directional cues from wound edges through a cellular monolayer undergoing collective migration, Zaritsky *et al* [61] quantified the mean directionality at different distances from the monolayer edge over time, followed by PCA. They were able to identify guanine nucleotide exchange factors (GEFs) that are involved in intercellular communication. Zhou *et al* [36] also developed a generalized computational framework, MOSES (motion sensing superpixels), to describe collective cell migration in terms of the individual trajectories of migrating cells and their spatiotemporal interactions (figure 2(j)). The long-term motion tracks from individual cells were constructed by capturing spatial motion dynamics with superpixels (formed with specific spatial points grouped with neighboring pixels at certain time points). The locations of superpixels in the next frame were calculated by averaging optical flow. Then, superpixels were linked to meshes to indicate the relationship between the movement of superpixels and that of their neighbors. Local and global spatiotemporal features can be derived from the strain curves, which measure the relative deformation between connected superpixels with respect to their initial mesh geometry over time (figure 2(k)). They applied PCA to the strain curves to visualize the phenotypic distributions of 2D collective migration (figure 2(l)). They demonstrated that the junctional motion dynamics of squamous-columnar cells with increasing epidermal growth factor (EGF) became similar to those of squamous-cancer cells. MOSES can provide a useful framework to investigate many biological collective phenomena in a quantitative and unbiased manner.

Different subcellular regions of leading edges are interconnected via cytoskeleton and membrane structures. Therefore, spatiotemporal feature-based phenotyping of leading-edge dynamics could provide additional insights in comparison to time-focused feature extraction. Ma *et al* [32] combined local shape descriptors and temporal features of time series for spatiotemporal feature extraction and studied the phenotypes of COS-7 cell edge dynamics. The cell edges were segmented and divided into sampling windows, and the time series of protrusion velocity was calculated for each window over time. They applied empirical mode decomposition, which reduces the data to six intrinsic mode functions (IMFs). The frequency spectra of each IMF in both the spatial and time domains were acquired by applying the Hilbert–Huang transform. Thereafter, they compiled instantaneous temporal and spatial frequency spectra into one feature vector for each time point and each sampling window. These features were used to merge similar neighboring spatial and temporal points by statistical region merging clustering and identify distinct motion regimens. They were able to locate subcellular microdomains with distinct Rac1 signaling activities.

### 4.2. Feature learning

Deep learning offers an entirely new approach for feature extraction. Instead of determining what kinds of features are useful for specific problems, DDNs are trained for certain tasks. The successful training of the neural networks means that the features learned by the neural networks can represent the input data very well in low dimensional space (figure 1(b)). This feature learning is an attractive method for feature extraction since it does not require prior assumptions about which features would be necessary for the problems. Even if the application of feature learning in morphodynamic phenotyping is still in its infancy, this is likely to accelerate morphodynamic phenotyping since it is very challenging to acquire sufficient prior information in regard to heterogeneous morphodynamic phenotypes. Although end-to-end training is known to be one of the advantages of deep learning, most of the feature learning for morphodynamic phenotyping involves multistage training. Usually, the morphological features are learned during the first stage of training. Then, using these features, different types of DL or conventional ML algorithms are used for temporal analyses.

Buggenthin *et al* [74] pioneered the application of DNNs to live cell images and demonstrated strong feature learning capability for stem cell differentiation. They trained a CNN to classify primary murine HSPCs into granulocytic/monocytic (GM) or megakaryocytic/erythroid (MegE) lineages to learn the static features from static brightfield microscopy images of differentiating primary hematopoietic progenitors. These CNN features along with cell movement were used to train another neural network, recurrent neural network [104] (RNN, a type of artificial neural network where previous output is used for current input) to forecast their lineage choice. They found that lineage choice can be detected before three generations using label-free live cell video when conventional fluorescence molecular markers are not observable. This means that DNNs are highly capable of capturing useful information in live cell video that cannot be detected by the human eye.

**Figure 3.**
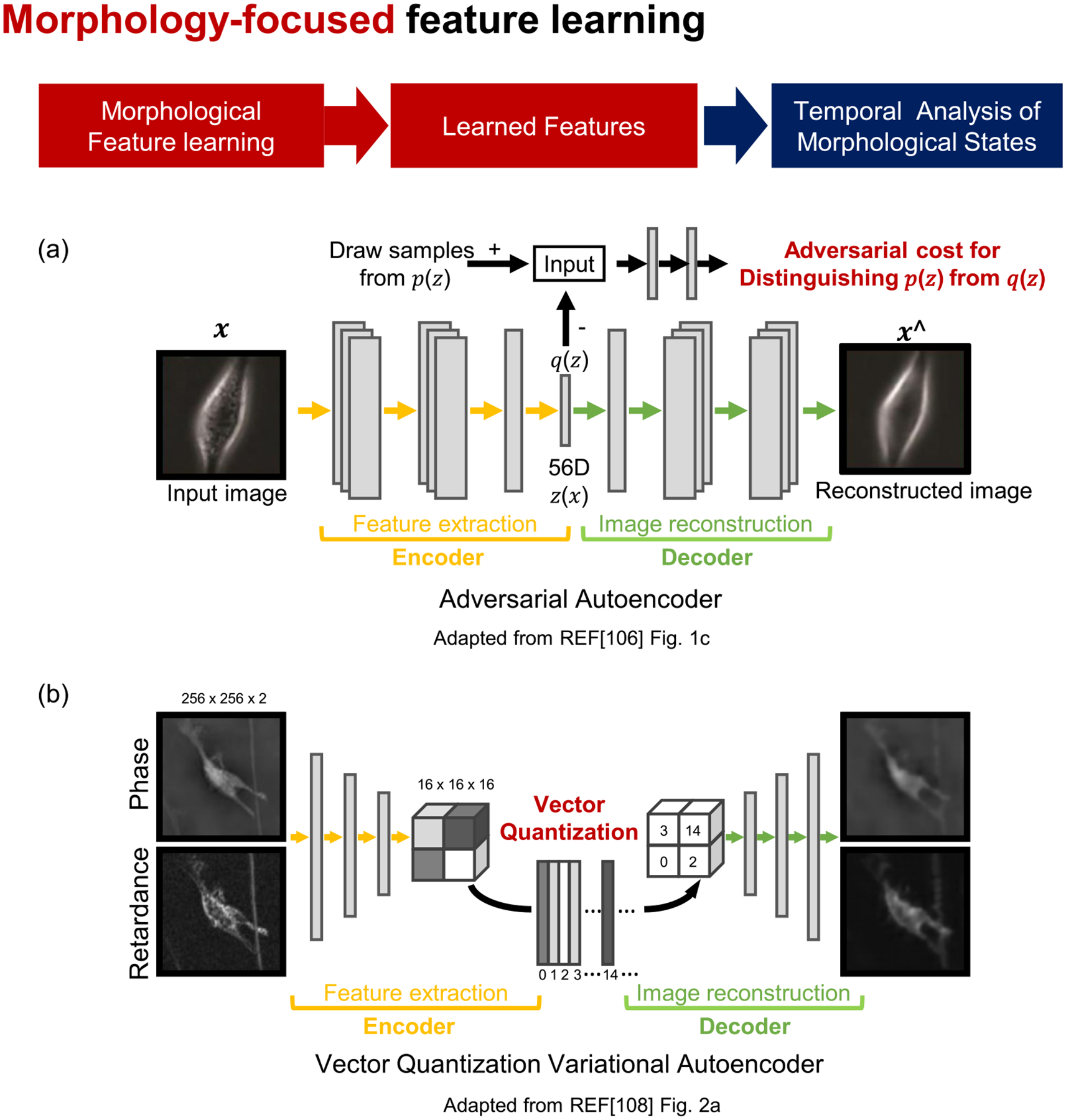
Phenotyping of cell morphodynamics based on morphology-focused feature learning. (a) and (b) Phenotyping procedure by morphology-focused feature learning. Autoencoders learn cellular morphological features. (a) Adversarial training and (b) vector quantization variational autoencoder. Panel (a) is adapted from figure 1(c) from reference [106]. Panel (b) is adapted from figure 2(a) from reference [108]. All panels are licensed under (CC-BY-4.0).

Generative adversarial networks (GANs, a generative modeling strategy where two artificial neural networks are trained to compete with each other) can automatically discover the patterns in input data and generate new data that resemble the original dataset [105]. Zaritsky *et al* [106] used a GAN variant, an adversarial autoencoder (figure 3(a)), to learn the morphological features of melanoma cells for cancer diagnosis. Then, the time-averaged morphological features were classified using linear discriminant analysis [107] (LDA, see appendix C) to predict the metastatic efficiency of patient-derived xenograft melanoma stage III cells. While deep learning features are usually not interpretable due to the inherent black-box nature of deep learning, they manipulated the autoencoder features to identify the interpretable cellular information that determines the aggressiveness of metastatic cells. This work demonstrated that deep learning analyses of label-free live cell images can be applied for cancer diagnosis.

Wu *et al* [108] developed a computational framework, DynaMorph, that employed deep learning for the automated discovery of morphodynamic states. The features of cell morphology were learned by training a CNN model using a vector quantized variational autoencoder (VQ-VAE, figure 3(b)). To understand the interplay between morphology and dynamic behavior, morphodynamic feature vectors were generated by combining the VQ-VAE morphological features, the trajectory-averaged principal components, and averaged displacements between frames. Unsupervised clustering was then performed to reveal novel morphodynamic states. This approach identified two distinct phenotypes of microglial cells that exhibit morphodynamically distinct responses upon immunological challenges. The authors further compared the morphodynamic states and gene expression patterns, demonstrating that the different morphodynamic phenotypes are correlated with differential gene expression programs.

In contrast to previous feature learning, Li *et al* [73] applied a CNN to simultaneously extract spatiotemporal features for cell dynamic morphology classification. The cell dynamic morphology in video data was converted into 2D image data by quantifying local edge displacement over time, and then the CNN was used to extract features to discover edge deformation patterns. They pursued a transfer learning approach, where the pretrained models (VGG16 or VGG19) were used for feature extraction, and SVM was used as a classifier. They demonstrated that their morphodynamic features were useful for classifying the immune activation of mouse lymphocytes.

## 5. Discussion

Cell populations have been widely observed to respond heterogeneously to molecular and environmental perturbations [33, 109, 110]. Poorly characterized cellular phenotypes make it challenging to interpret the true observed heterogeneity. Furthermore, today, with the increased volume of resultant datasets along with the development of imaging and genomic technologies, dissecting heterogeneity in cellular datasets under specific experimental conditions faces many challenges for the identification of subtle but significant phenotypic variations in cellular dynamics.

The task of assessing cellular dynamic states requires a high-throughput and fully automated approach to analyze a massive amount of data for statistically significant discrimination to determine rare but biologically meaningful dynamic cellular phenotypes. However, the longitudinal monitoring of dynamic cellular responses with live-cell imaging remains a low-throughput endeavor. High-throughput studies with long-term and large-scale examinations of cell populations, including neuronal differentiation [111] and cell lineages of *S*. *pombe* [112], are usually limited in low-resolution settings. Conventional microscopes cannot acquire high-resolution and large field-of-view images at the same time. Therefore, the resolution enhancement of live cell imaging seeks to address this challenge to advance the identification of detailed motility and morphodynamic phenotypes. Fourier ptychographic microscopy [113] can stitch the Fourier components from low-resolution and a large field of view images with different directions of illumination, generating high-resolution large field-of-view images. DL can also improve image resolution by training neural networks to translate low-resolution to high-resolution microscopy images [114]. These computational imaging technologies will likely make significant contributions to the development of high-throughput live cell imaging and the integration of cellular motility and morphodynamics across various spatial and temporal scales.

Recently implemented multiomics measurements of genomes, transcriptomes, epigenomes, proteomes, and chromatin organization have opened up new avenues to disentangle the causal relationship between multiomics layers and cellular phenotypes. Integrating these multiple datasets will provide more comprehensive phenotypes [115]. While spatial transcriptomics integrating single-cell RNA-seq and static cellular image data is emerging [116, 117], significant technical hurdles remain for integrating live cell imaging with multiomic technologies. It is expected that MERFISH [118], revealing the spatial distribution of hundreds to thousands of RNA species in individual cells, could be employed for this purpose in the future.

The comprehensive identification of biologically meaningful phenotypes hidden in phenotypic heterogeneity remains a major goal. Many fine-grained dynamic phenotypes can arise from diverse live cell images and multiomic datasets in conjunction with new ML analysis techniques. Given that cellular motility and morphodynamics reflect the states of cellular physiology and pathophysiology, this effort will allow us to deconvolve their heterogeneity and uncover molecular and cellular mechanisms of disease progression in unprecedented detail. This will ultimately open up fresh opportunities for live cell-based drug discovery and diagnosis.

## Figures and Tables

**Figure 4.**
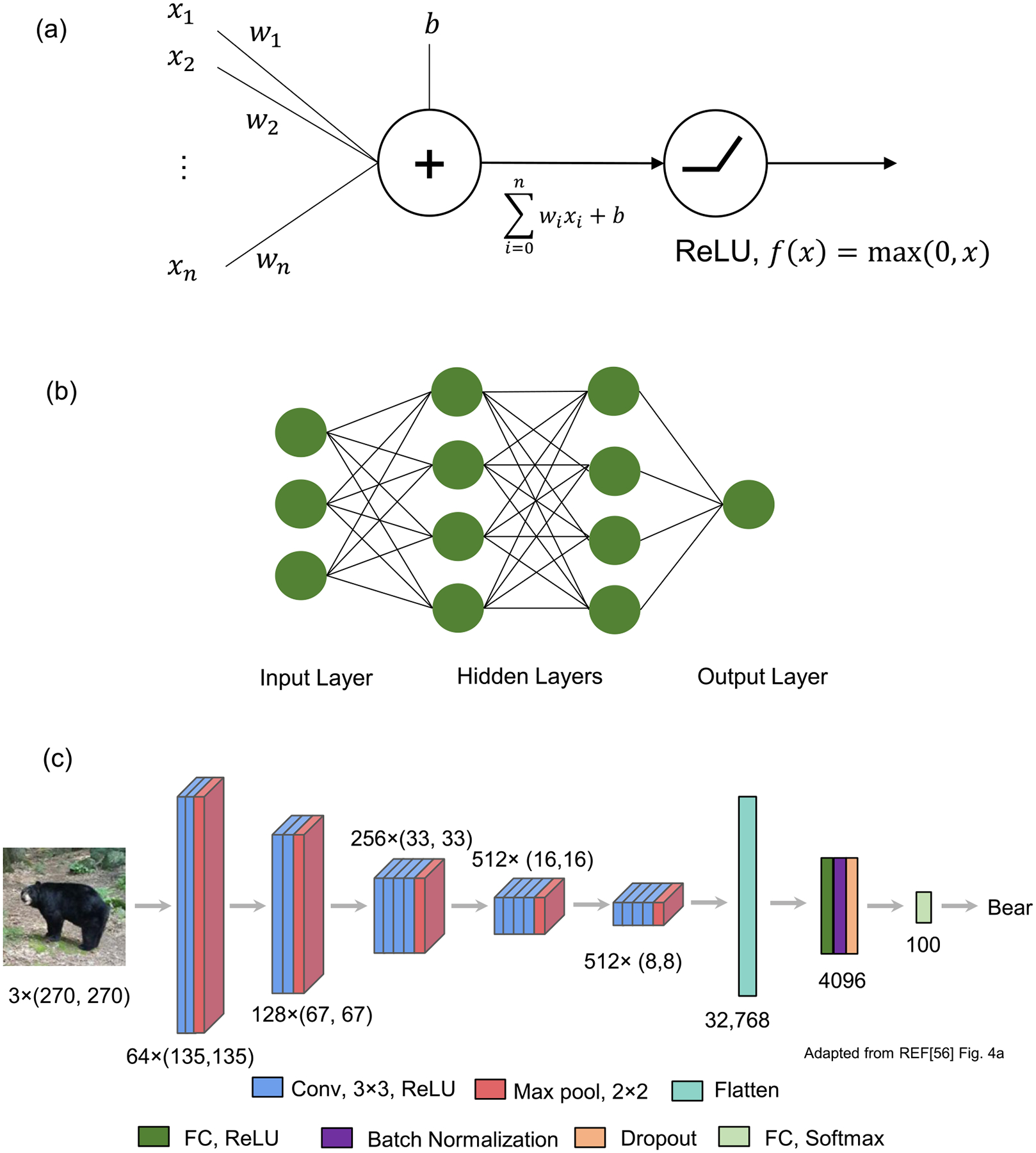
Deep learning models. (a) A mathematical model of a neuron. The weighted sum of input values is transformed by a nonlinear activation function. A ReLU (rectified linear unit) is a widely used activation function. (b) Fully connected artificial neural network. (c) An example of a CNN for image classification. Conv, 3 × 3, ReLU: convolutional layer with 3 × 3 filters and ReLU activation. Max pool, 2 × 2: max pooling layer with 2 × 2. FC: fully connected layer. (a) × (b) and (c): output format of a convolutional layer ((a): the number of filters, (b) × (c): the size of filter image). Panel (c) is adapted from figure 4(a) in reference [56] and licensed under (CC-BY-4.0).

## Data Availability

No new data were created or analysed in this study.

## Appendix A. Phenotyping

‘Phenotype’ originates from the Greek words *phainein* (meaning ‘shining’ or ‘to show’) and *typos* (meaning ‘type’). A phenotype is defined as the observable properties of an organism, including but not limited to behavior, biochemical properties, color, shape, and size, that result from both its genotype (total genetic inheritance) and the external environment. Biological phenotypes differ from the characteristics of nonliving or inanimate objects in two main ways: (1) they are innately inherited, and (2) they continuously evolve over generations [119, 120] such that they exhibit plasticity during development [121] or as a way of adapting to their environment [122]. As the broad definition of the terminology suggests, the meaning of the word continues to evolve given the perpetual technological evolution of today’s observational tools. In addition, our understanding of biological systems has evolved in an effort to elucidate the mechanisms that underlie the generation of highly complex naturally occurring biological phenotypes. In contrast to a phenotype, a genotype is defined as the genetic composition of an individual organism. Elucidating the mechanism by which genotypes generate phenotypes and how the environment affects the relationship between genotypes and phenotypes is still a major challenge in biology, both for basic research and for practical applications in medicine and agriculture.

## Appendix B. Deep learning

Deep learning is a technique that uses a network of neuron-like components to learn the input and output relationships from the data [104, 123–125]. Since 1943, it has evolved gradually from a mathematical model (perceptron) that mimics a neuron in the brain [126]. Each neuron can take multiple inputs from other neurons and output a value to another neuron with nonlinearity (figure 4(a)). Every connection between neurons has a weight or parameter that is updated during the training process. Together, they form an artificial neural network, minimally resembling neural networks in brains (figure 4(b)) [127]. With the advent of powerful GPUs (graphics processing units) and large-scale image databases, artificial neural networks with more than three layers, called DNNs, have opened a new era of artificial intelligence [124].

The strength of deep learning is that a DNN can approximate any function. In other words, it can map or find the relationship between the input and output of any data [128]. For instance, a DNN can take an image as input and output the name of the object in the image [124]. As a result, deep learning methods are applied in various ways, such as image recognition [124] and speech recognition [129], protein folding [130], drawing art [131], and playing Go games [132]. The high dimensionality of a DNN allows it to excel at learning patterns or features from the high dimensional data of the real world [133]. However, this property also makes the DNN easily overfit to training data, where a DNN learns the patterns specific to training data, which do not exist in unseen real-world testing data. The best way to prevent overfitting is to use a large amount of training data [123]. Training datasets, however, are not always large due to high cost and time. Therefore, several regularization techniques, such as data augmentation [124], dropout [134], and batch normalization [135], have been employed to mitigate overfitting.

CNNs are the most popular type of deep neural network widely used in computer vision tasks that extract information from the image and understand its content (figure 4(c)) [104, 123]. The CNN architecture used for image classification comprises a sequence of convolutional and pooling layers with fully connected layers at the end [124]. Each convolutional layer contains a block of neurons, called a filter, that finds local spatial patterns from the two-dimensional (2D) input and outputs a 2D feature map. Then, the pooling layer summarizes neighboring values and reduces the feature map to a lower resolution so that the next convolutional layers can detect features at a higher receptive field. Fully connected layers flatten the feature map to the 1D vector and learn to output numbers representing the classes of input images. This hierarchical structure of the CNN that processes from low- to higher-level features resembles how our brains process visual information [127, 136].

## Appendix C. Machine learning glossary

- Autocorrelation function (ACF) [99]: a common time series feature that quantifies the similarity between a time series and its lagged one.
- Autoencoder (AE) [49]: a type of artificial neural network widely used for feature learning. It reproduces the input of the AE with a reduced number of hidden units, whose values can serve as the learned features from the data.
- CNNs [104]: the most popular type of artificial neural network used for image data. They automatically learn multilevel image features for image classification.
- Density-peak clustering [100]: a clustering algorithm that identifies cluster centers that are at local density maxima and away from other high-density regions.
- Generative adversarial network (GAN) [105]: a generative modeling strategy where two artificial neural networks (generator and discriminator) are trained to compete with each other.
- Haralick feature [95]: a feature that quantifies texture information from images.
- Hidden Markov model (HMM) [92]: a statistical technique that models observed data by the probabilistic transitions of hidden states by assuming a Markov process.
- K-means [97]: a clustering algorithm that groups unlabeled data into *k* clusters by assigning them to nearest cluster means (centroids).
- Linear discriminant analysis (LDA) [107]: an algorithm that finds a linear combination of features that separates given classes of data.
- Principal component analysis (PCA) [89]: an algorithm that reduces the dimensionality of data while preserving the variance of data as much as possible.
- Recurrent neural network (RNN) [104]: a type of artificial neural network where previous output is used for current input. It is widely used for time series modeling and natural language processing.
- Support vector machine (SVM) [91]: a classifier that maximizes the space between two classes.
- t-distribution stochastic neighbor embedding (t-SNE) [96]: assigning pairs of similar data with high probabilities of neighbors in low-dimensional space.
